# High tumor hexokinase-2 expression promotes a pro-tumorigenic immune microenvironment by modulating CD8+/regulatory T-cell infiltration

**DOI:** 10.1186/s12885-022-10239-6

**Published:** 2022-11-01

**Authors:** Sehui Kim, Jaemoon Koh, Seung Geun Song, Jeemin Yim, Miso Kim, Bhumsuk Keam, Young Tae Kim, Jihun Kim, Doo Hyun Chung, Yoon Kyung Jeon

**Affiliations:** 1 Department of Pathology, Seoul National University College of Medicine, 103 Daehak-ro, Jongno-gu, 03080 Seoul, Republic of Korea; 2 Department of Pathology, Yonsei University College of Medicine, Seoul, Republic of Korea; 3 Department of Biomedical Sciences, Seoul National University College of Medicine, Seoul, Republic of Korea; 4 Department of Internal Medicine, Seoul National University College of Medicine, Seoul, Republic of Korea; 5 Department of Thoracic Surgery, Seoul National University Hospital, Seoul National University College of Medicine, Seoul, Republic of Korea; 6 Seoul National University Cancer Research Institute, Seoul, Republic of Korea; 7 Department of Pathology, Asan Medical Center, University of Ulsan College of Medicine, Seoul, Republic of Korea

**Keywords:** Hexokinase-2, Glycolysis, Tumor microenvironment, Tumor-infiltrating lymphocytes, CD8 + T-cell to Treg ratio, Immunotherapy

## Abstract

**Background:**

Relationship between cancer cell glycolysis and the landscape of tumor immune microenvironment in human cancers was investigated.

**Methods:**

Forty-one fresh lung adenocarcinoma (ADC) tissues were analyzed using flow cytometry for comprehensive immunoprofiling. Formalin-fixed tissues were immunostained for hexokinase-2 (HK2) to assess cancer cell glycolysis. For validation, formalin-fixed tissues from 375 lung ADC, 118 lung squamous cell carcinoma (SqCC), 338 colon ADC, and 78 lung cancer patients treated with anti-PD-1/PD-L1 immunotherapy were immunostained for HK2, CD8, and FOXP3.

**Results:**

Based on immunoprofiling of lung ADC, HK2 tumor expression was associated with the composition of lymphoid cells rather than myeloid cells. High HK2 tumor expression was associated with immunosuppressive/pro-tumorigenic features, especially decreased ratio of CD8 + T-cells to Tregs (rho = −0.415, *P* = 0.012). This correlation was also confirmed in four different cohorts including lung ADC and SqCC, colon ADC, and the immunotherapy cohort (rho = −0.175~-0.335, all *P* < 0.05). A low CD8 + T-cell to Treg ratio was associated with poor progression-free survival and overall survival in lung SqCC patients, and a shorter overall survival in the immunotherapy cohort (all, *P* < 0.05).

**Conclusion:**

An increase in HK2 expression may contribute to shaping the immunosuppressive/pro-tumorigenic tumor microenvironment by modulating the CD8 + T-cell to Treg ratio. Targeting tumor HK2 expression might be a potential strategy for enhancing anti-tumor immunity.

**Supplementary Information:**

The online version contains supplementary material available at 10.1186/s12885-022-10239-6.

## Background

Cancer immunotherapy targeting the PD-1/PD-L1 pathway is clinically beneficial and widely used in patients with a variety of cancers [1–3]. However, only a small proportion of patients show a good response to immunotherapy; thus, there have been many efforts to uncover predictive biomarkers to improve PD-1/PD-L1 blockade [4]. PD-L1 expression and the tumor mutational burden in lung cancer are used as predictive biomarkers in clinical practice but have limited predictability [5–7]. This may be attributed to the complexity of the tumor microenvironment (TME), since the responsiveness to immunotherapy is affected by the tumor immune microenvironment [8]. In general, a pro-inflammatory microenvironment rather than an immunosuppressive microenvironment predicts a good response to immunotherapy.

Tumor and TME crosstalk occurs via diverse genetic and non-genetic factors, which shape the tumor immune microenvironment [9]. For example, β-catenin signaling, RHOA mutation, PTEN loss, LKB1 mutation, and KRAS mutations were associated with an immunosuppressive microenvironment, which included reduced CD8 + T-cell infiltration and increased regulatory T-cell (Treg) infiltration [9–14]. In addition, the metabolic status of tumor cells can affect immune cell infiltration and function [15, 16].

Within the tumor niche, tumor cells and surrounding environmental cells, especially immune cells, compete for limited nutrients and oxygen. Therefore, tumor metabolism can affect tumor-infiltrating immune cells. Because tumor cells predominantly use glycolysis for their survival and proliferation [17], immune cells often encounter low glucose and high lactic acid levels in the TME. Previous studies demonstrated that high tumor glycolysis results in an immunosuppressive TME by reducing effector T-cell functions [15, 16]. However, the effects of tumor glycolysis on other immune cells remain unclear.

Each subset of immune cells in the TME utilizes different metabolic programs for their survival, activation, and differentiation [18, 19]. Effector T-cells are highly glycolytic, but Tregs depend on oxidative phosphorylation (OXPHOS) and fatty acid oxidation (FAO) [20–23]. Activated dendritic cells use glycolysis predominantly, and pro-inflammatory tumor-associated macrophages (TAMs) are highly glycolytic. However, tolerogenic TAMs usually use oxygen-driven metabolism, such as OXPHOS and FAO [24]. Tumor-infiltrating myeloid-derived suppressor cells (MDSCs) in mice prefer FAO over glycolysis as a primary source of energy [25]. Therefore, each immune cell subset might be uniquely affected depending on the type of tumor metabolism.

Previously, we evaluated tumor HK2 expression, as a marker of glycolysis and analyzed the relationship between tumor HK2 expression and T cell function/infiltration. Previous study showed that lung cancer cell HK2 overexpression suppressed T-cell effector functions, and that the increased HK2 expression was inversely correlated with the expression of T-cell effector molecules according to analysis of The Cancer Genome Atlas in lung cancer [16]. However, little is known about the landscape of the tumor immune microenvironment in the context of tumor glycolysis in human cancer tissues. Thus, we comprehensively investigated the relationship between tumor glycolysis and tumor-infiltrating immune cells in human cancer tissues. We demonstrated that a higher HK2 expression which stands for higher rate of tumor glycolysis is associated with an immunosuppressive microenvironment characterized by a decrease in CD8 + T-cell infiltration relative to Treg infiltration in lung and colon cancers.

## Materials and methods

### Patients and samples

Fresh tumor samples from 41 lung adenocarcinoma (ADC) patients who underwent surgery at Seoul National University Hospital (SNUH) were used for flow cytometry analysis. All patients agreed to the sample collection and data analyses. Written informed consent was provided for this cohort. For immunohistochemistry (IHC) analysis, tissue microarrays were constructed from formalin-fixed paraffin-embedded (FFPE) tumor tissues from the above 41 lung ADC patients, and an additional 375 lung ADC and 118 lung squamous cell carcinoma (SqCC) patients from SNUH and 338 colon ADC patients from Asan Medical Center. Additionally, 78 non-small cell lung cancer patients treated with PD-1/PD-L1 blockade at SNUH were included for further validation. Informed consent for participation in the IHC analysis only was waived by the SNUH and Asan Medical Center institutional review boards because this was a retrospective study using archived material and did not increase patient risk. The tumor, node, and metastasis staging system was performed based on the 7th American Joint Committee on Cancer (AJCC) for lung cancer and the 8th AJCC for colon cancer [26, 27]. Clinicopathological features of patients are summarized in Supplementary Tables S1, 2, 3, 4 and 5. This study followed the World Medical Association Declaration of Helsinki recommendations and was approved by the institutional review board of SNUH (No.: H-1404-100-572 and H-1905-115-1035).

### Flow cytometry analysis

To analyze tumor-infiltrating immune cell subsets, 1 g fresh lung ADC tissues were subjected to flow cytometric analysis, as described previously [28]. Data were analyzed using FlowJo v10.1 software (Treestar), and the gating strategy is shown in Supplementary Figure S1.

### Immunohistochemistry (IHC)

IHC was performed using the following antibodies: rabbit anti-HK2 polyclonal antibody (Genetex, Irvine, CA, USA), rabbit anti-CD8 monoclonal antibody (clone SP16, Thermo Fisher Scientific, Rockford, IL, USA) and rabbit anti-FOXP3 monoclonal antibody (236 A/E7, Abcam, Cambridge, UK). Immunostaining was performed using the Benchmark XT autostainer (Ventana Medical Systems, Tucson, AZ, USA). The H-score of HK2 was calculated as follows: sum of each staining intensity (0−3) ⋅ proportion (0−100%). The numbers of CD8 + and FOXP3 + tumor-infiltrating lymphocytes (TILs) per mm^2^ were automatically counted via modified nuclear IHC algorithms using Aperio ImageScope software (Aperio Technologies, Vista, CA, USA).

### Statistical analyses

The correlation between tumor HK2 expression and tumor-infiltrating immune cells was calculated using Spearman’s correlation. Two-sided *P* values < 0.05 were considered statistically significant. The cutoff values of the variables were determined by receiver operating characteristic curve analysis. Survival analysis was performed using Kaplan−Meier analysis and the log-rank test. Two-sided *P* values < 0.05 were considered statistically significant in all analyses. All statistical analyses were performed using R statistical software 4.1.1 (R Foundation for Statistical Computing, Vienna, Austria). Images were created using the GraphPad Prism 7 software.

## Results

### Tumor glycolysis is differentially associated with each subset of immune cells

**Fig. 1:**
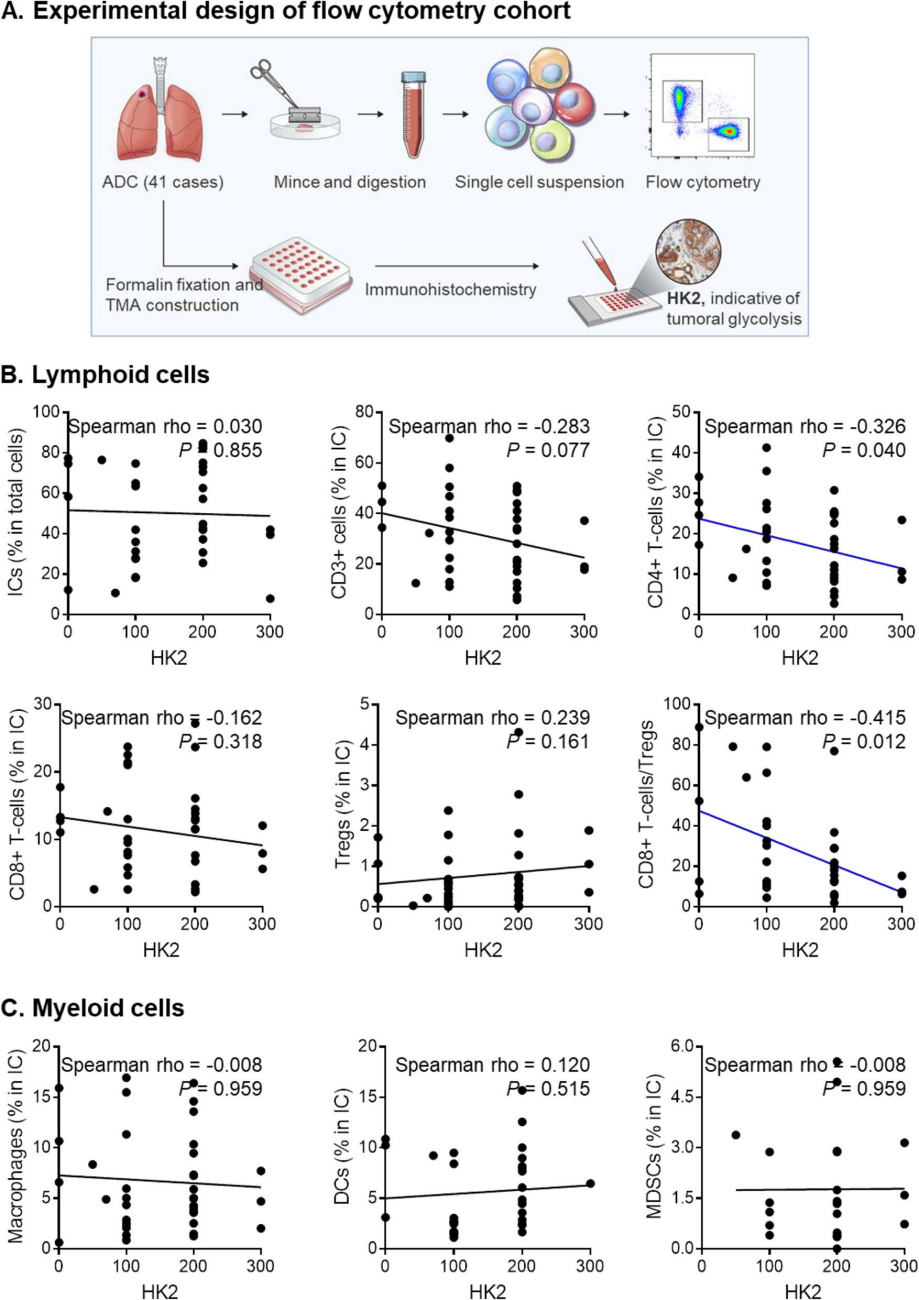
The relationship between tumor HK2 expression and tumor-infiltrating immune cell subsets analyzed by flow cytometry. **A** Flow cytometry experimental design. **B** The total immune cell and lymphoid cell infiltration statuses according to tumor HK2 expression are depicted. **C** Myeloid cell infiltration according to tumor HK2 expression is depicted. All *P* values were calculated using Spearman correlation analysis

Using flow cytometry, tumor-infiltrating lymphoid cells and myeloid cells from lung ADC fresh tissues from 41 patients were comprehensively profiled (Supplementary Figure S1). IHC was used to evaluate HK2 expression in tumor cells, as an indicator of tumor glycolysis, because HK2 is the first and the rate−limiting enzyme of glycolysis. The experimental scheme is described in Fig. 1 A, and the correlations between individual immune cell subsets and tumor HK2 expression are summarized in Fig. 1B, C; Table 1.

**Table 1:** Correlations between tumor HK2 expression and immune cell subsets

Immune cell subsets	Correlation coefficient^a^	*P* value
Pan-immune cells (% of total cells)	0.030	0.855
CD3 + cells (% of ICs)	−0.283	0.077
CD4 + cells (% of CD3 + cells)	−0.225	0.162
CD4 + cells (% of ICs)	−0.326	0.040
CD8 + cells (% of CD3 + cells)	0.114	0.485
CD8 + cells (% of ICs)	−0.162	0.318
Treg (% of CD4 + cells)	0.480	0.003
Tregs (% of ICs)	0.239	0.161
CD8 + cells to Tregs ratio	−0.415	0.012
CD19 + cells (% of ICs)	−0.377	0.017
NK cells (% of ICs)	−0.134	0.411
Macrophages (% of ICs)	0.023	0.888
M0 (% of macrophages)	−0.116	0.477
M1 (% of macrophages)	0.089	0.584
M2 (% of macrophages)	−0.010	0.949
DCs (% of ICs)	0.091	0.604
MDSCs	0.028	0.901

*Abbreviations*: *IC* Immune cell, *Tregs* Regulatory T-cells, *DC* Dendritic cell, *MDSC* Myeloid-derived suppressor cell

^a^Spearman correlation analysis

Total immune cell infiltration did not differ when quantified according to tumor HK2 expression. Tumor HK2 expression was more closely related to lymphoid cells compared with myeloid cells (Fig. 1B, C). The proportion of CD3 + cells to total immune cells and CD4 + T-cells to total immune cells tended to be inversely correlated with HK2 tumor expression (spearman rho = −0.283, *P* = 0.077; spearman rho = −0.326, *P* = 0.040, respectively). The proportion of CD19 + B-cells to total immune cells was also inversely correlated with HK2 tumor expression (spearman rho = −0.377, *P* = 0.017; Table 1). Although the proportion of Tregs to total immune cells did not significantly differ according to tumor HK2 expression, the proportion of Tregs to CD4 + T-cells showed a significant positive correlation with tumor HK2 expression (spearman rho = 0.480, *P* = 0.003). Of note, the ratio of CD8 + T-cell to Tregs was inversely correlated with tumor HK2 expression (spearman rho = −0.415, *P* = 0.012) (Fig. 1B). Proportions of tumor-infiltrating macrophages, dendritic cells, and MDSCs did not differ according HK2 tumor expression (Fig. 1 C). In addition, HK2 tumor expression did not affect M1 versus M2 polarization of macrophages (Table 1). Among the above differentially infiltrating immune cells, we further validated the association between tumor HK2 expression and CD8 + T-cells, Tregs, and their ratio in additional larger cohorts.

### Tumor HK2 expression was inversely correlated with the ratio of CD8 + T-cells to Tregs

**Fig. 2:**
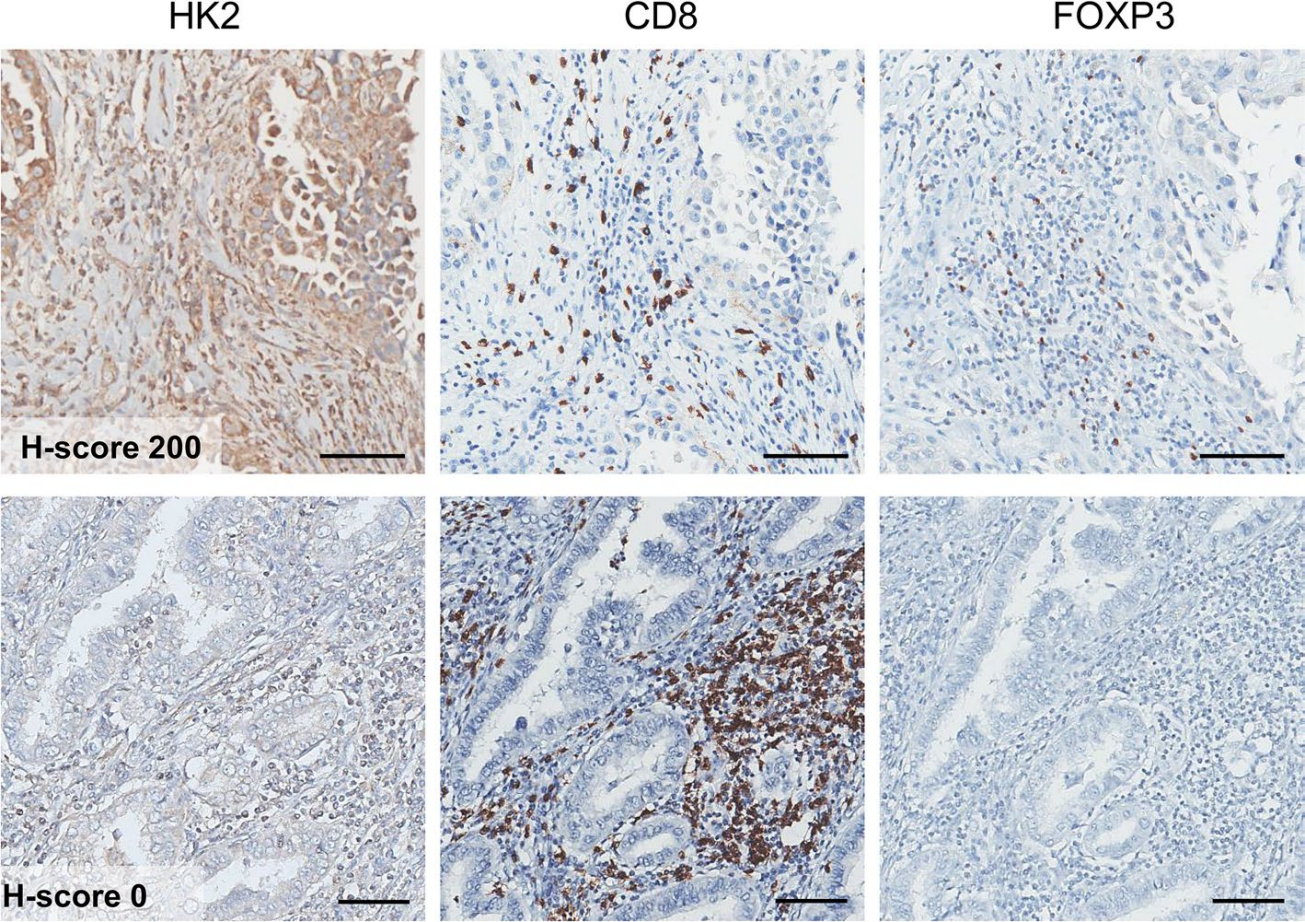
Representative images of HK2 tumor expression relative to the CD8 + T-cell to Treg ratio from lung adenocarcinoma cases. **A** High tumor HK2 expression with a low CD8 + T-cell to Treg ratio and (**B**) low tumor HK2 expression with a high CD8 + T-cell to Treg ratio. (Original magnification: ⋅200, bar = 100 μm)

**Fig. 3:**
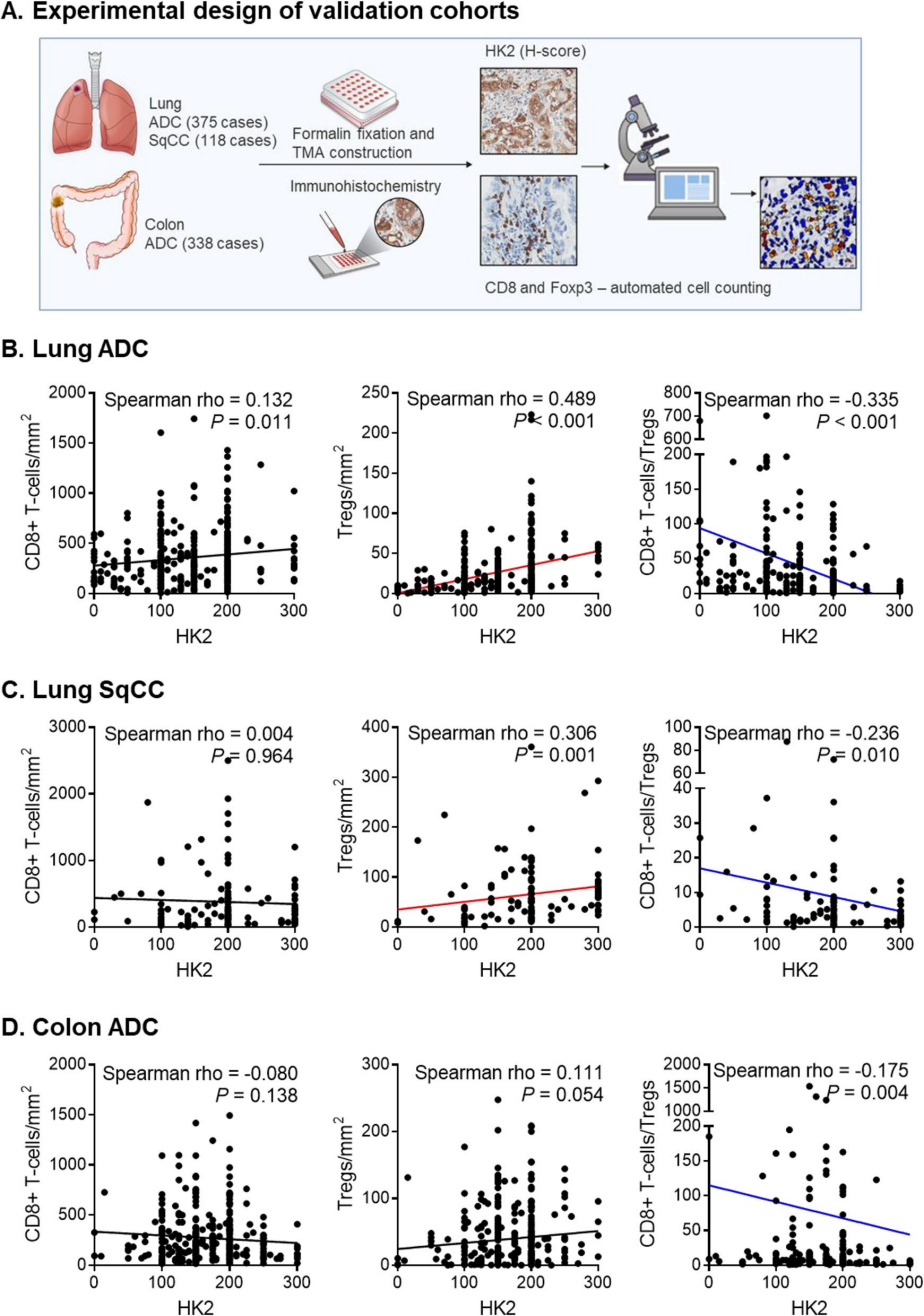
Correlation between HK2 tumor expression and immune cell composition including CD8 + T-cells, Tregs, and CD8 + T-cell to Treg ratio. **A** Experimental design of the validation cohorts. Results from 375 lung ADC patients (**B**), 118 lung SqCC patients (**C**), and 338 colon ADC patients (**D**) are depicted. All *P* values were calculated using Spearman correlation analysis

To validate the above findings, we evaluated tumor HK2, CD8 + cells, and Tregs (FOXP3 + cells) using IHC in 375 lung ADC, 118 lung SqCC, and 338 colon ADC cases (Fig. 2), as schematically described in Fig. 3 A. HK2 expression was correlated with some clinicopathologic parameters with marginal statistical significance (Supplementary Table S6, 7 and 8). In lung adenocarcinoma, EGFR mutant tumor tended to show lower HK2 expression than EGFR wild type tumor (*P* = 0.079). In colon adenocarcinoma, tumors with higher T stage tended to have higher HK2 expression than tumor with lower stage (*P* = 0.07) and when categorizing tumors T1-3 versus T4, T4 tumors showed statistically significant HK2 overexpression, compared to T1-3 tumors (*P* = 0.015, data not shown). The relationships of tumor HK2 expression with CD8 + T-cells, Tregs, and their ratio are summarized in Fig. 3B**−**D. CD8 + T-cell infiltration was not significantly correlated with tumor HK2 expression. In contrast, Treg infiltration was positively correlated with tumoral HK2 expression in lung adenocarcinoma and squamous cell carcinoma cohort (lung ADC, spearman rho = 0.489, *P* < 0.001; lung SqCC, spearman rho = 0.306, *P* = 0.001), and tended to be positively correlated with tumoral HK2 expression in colon adenocarcinoma cohort (spearman rho = 0.111, *P* = 0.054) (Fig. 3B-D). Of note, the ratio of CD8 + T-cells to Tregs was inversely correlated with HK2 tumor expression (lung ADC, spearman rho = −0.335, *P* < 0.001; lung SqCC, spearman rho = −0.236, *P* = 0.010; colon ADC, spearman rho = −0.175, *P* = 0.004) (Fig. 3B-D). These findings were consistent with the flow cytometry analyses and demonstrated that an increase in tumor glycolysis as represented by HK2 tumor expression was inversely correlated with the ratio of CD8 + T-cells to Tregs in patients with lung cancer and colon cancer.

### A lower CD8 + T-cell to Treg ratio was associated with poor survival in patients with lung SqCC

Whereas high CD8 + T-cell infiltration generally predicts a favorable prognosis and good response to immunotherapy in cancer patients, high Treg infiltration was not consistent in predicting the prognosis of patients [29]. Treg recruitment partially depends on the presence of CD8 + T-cells [30]. A significant positive correlation between Treg and CD8 + T-cell infiltration in lung and colon cancer cohorts was consistently observed (all *P* < 0.001) (Supplementary Figure S2).

**Fig. 4:**
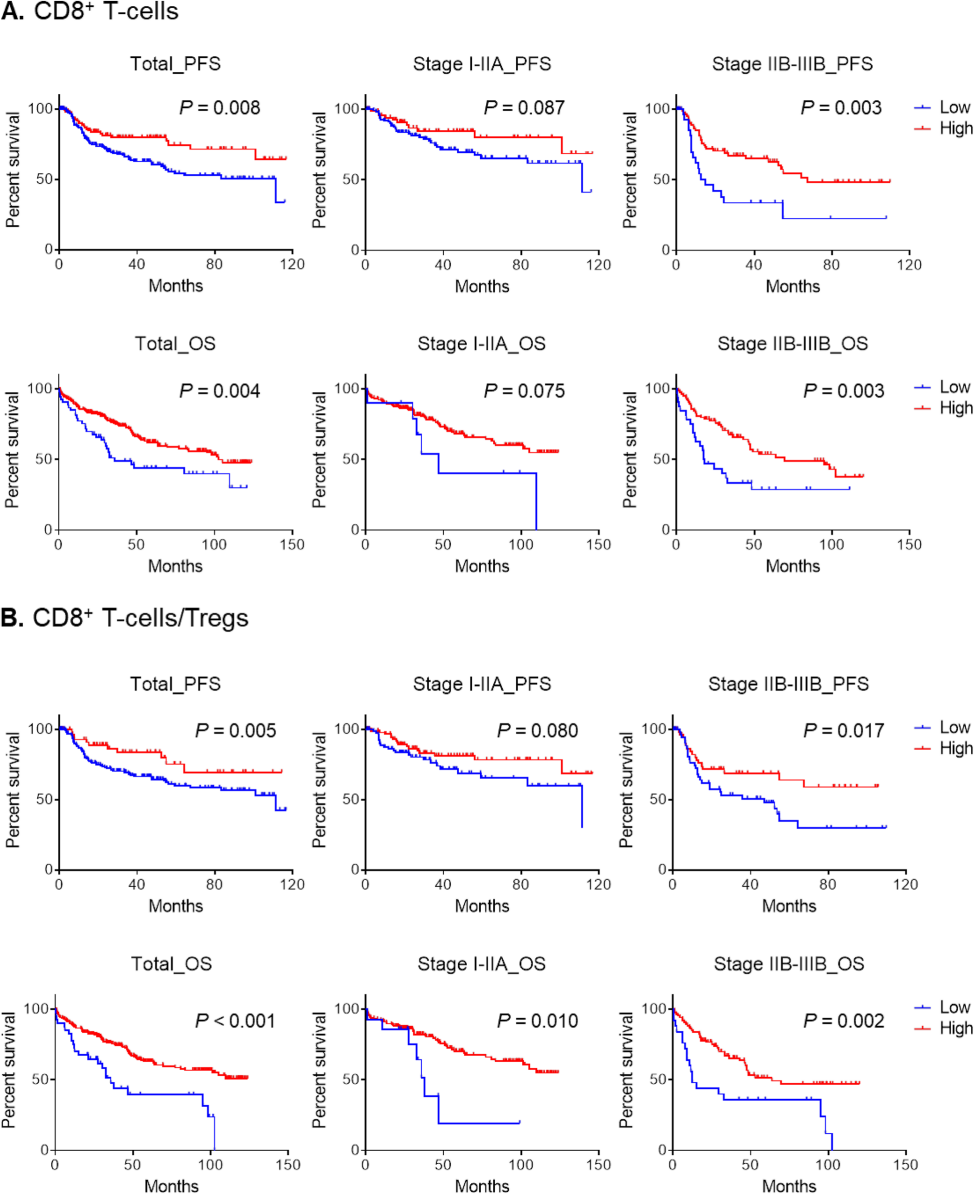
Survival analysis of patients with lung SqCC according to the number of CD8 + T-cells and the CD8 + T-cell to Treg ratio. **A** The progression-free survival (PFS) and overall survival (OS) according to the number of CD8 + T-cells are displayed in total, early-stage (stage I−IIA), and advanced-stage (stage IIB−IIIB) disease patients. **B** The PFS and OS according to the ratio of CD8 + T-cells to Tregs are displayed in the total, early-stage (stage I−IIA), and advanced-stage (stage IIB−IIIB) disease patients. The survival difference was plotted and analyzed using the Kaplan-Meier and log-rank test

We also evaluated the prognostic significance of the CD8 + T-cell to Treg ratio as well as the numbers of CD8 + T-cells and Tregs. In lung SqCC, a lower number of CD8 + TILs was associated with poor progression-free survival (PFS) and overall survival (OS) (*P* = 0.008 and *P* = 0.004, respectively) (Fig. 4 A). Of note, a lower ratio of CD8 + T-cells to Tregs was associated with poor PFS and OS (*P* = 0.005 and *P* < 0.001, respectively) **(**Fig. 4B**)**. A lower number of CD8 + TILs was associated with poor OS in patients with advanced-stage (IIB−IIIB) (*P* = 0.003) rather than early-stage (I−IIA) (*P* = 0.075) cancer (Fig. 4 A). In contrast, a decreased ratio of CD8 + T-cells to Tregs was associated with poor OS in patients with both early and advanced stages (*P* = 0.010 and 0.002, respectively). These findings suggest that the ratio of CD8 + T-cells to Tregs might be a more useful prognostic factor compared with CD8 + TILs. Together, these findings suggest that tumor HK2 expression influences patient survival indirectly via distinct immune profiling, specifically a low ratio of CD8 + T-cells to Tregs.

### Implication of the CD8 + T-cell to Treg ratio in patients treated with immunotherapy

**Fig. 5:**
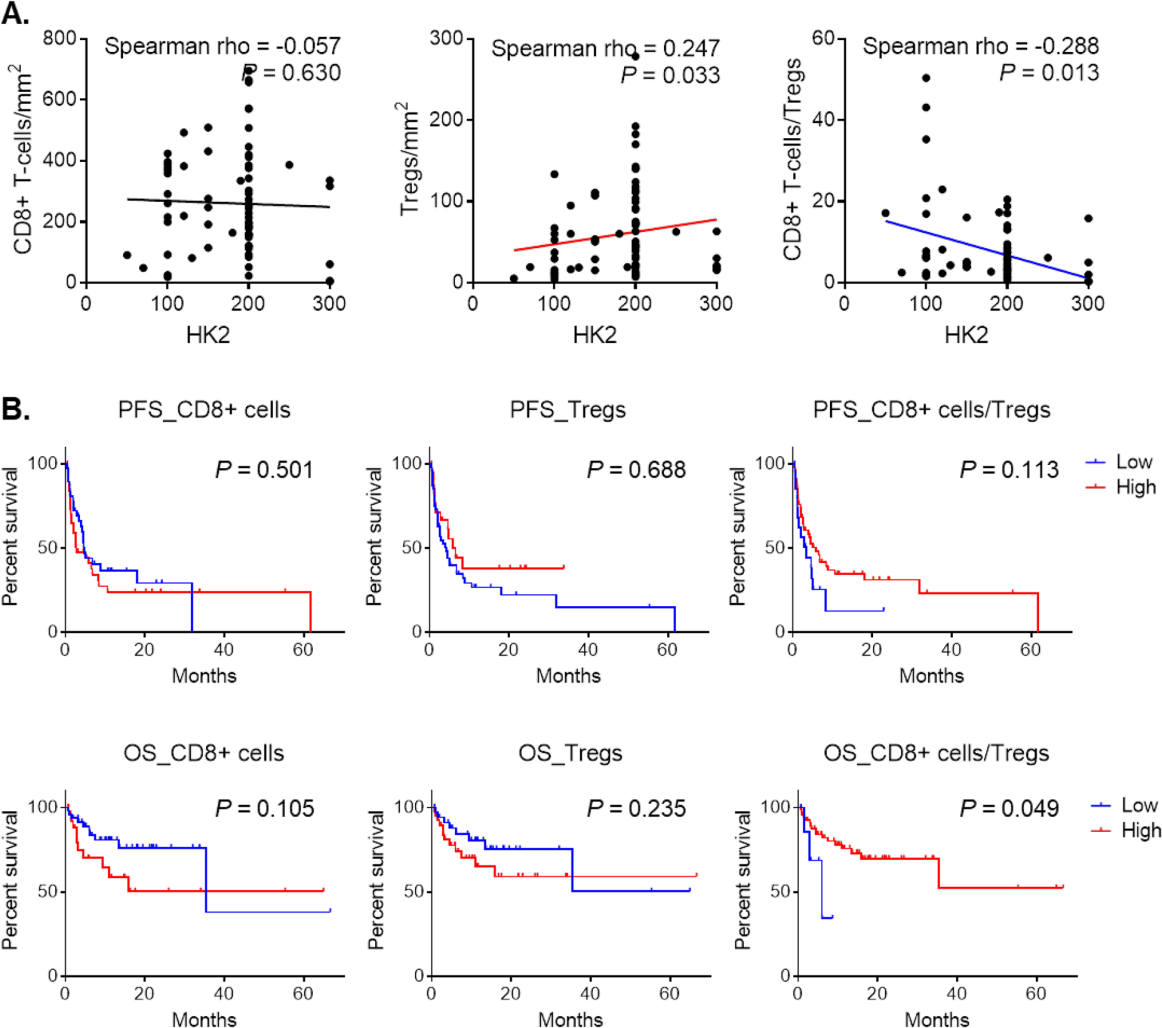
The correlation between HK2 tumor expression and immune cell composition or survival analysis according to immune cells in non-small cell lung cancer patients treated with PD-1/PD-L1 immunotherapy. **A** The correlation between tumor HK2 expression and immune cell composition including CD8 + T-cells, Tregs, and the CD8 + T-cells to Treg ratio. **B** Survival analysis of patients according to the number of CD8 + T-cells and the CD8 + T-cell to Treg ratio

An additional validation cohort that included 78 non-small cell lung cancer patients with PD-1/PD-L1 blockade therapy was also investigated. Treg infiltration, but not CD8 + T-cell infiltration, was positively correlated with HK2 tumor expression (spearman rho = 0.247, *P* = 0.033). The CD8 + T-cell to Tregs ratio was also inversely correlated with HK2 tumor expression (spearman rho = −0.288, *P* = 0.013) (Fig. 5 A). The CD8 + T-cell to Tregs ratio was a better discriminator for prognosis compared with CD8 + T-cells and Tregs individually. Additionally, patients with a high CD8 + T-cell to Treg ratio tended to show prolonged PFS and OS after immunotherapy (*P* = 0.113 and 0.049, respectively) (Fig. 5B).

## Discussion

In this study, HK2 tumor expression as an indicator of glycolysis was associated with a different immune profile in the TME. Additionally, the present T-cell subsets within the TME significantly differed according to the level of HK2 tumor expression. As described previously, each subset of immune cells relies on different metabolic pathway for their survival, activation, and differentiation.

CD4 + helper and CD8 + T-cells rely on glycolysis and mammalian target of rapamycin (mTOR) signaling for their metabolic activity. In contrast, Tregs rely on FAO and OXPHOS, and mTOR suppresses differentiation into Tregs [22, 31, 32]. Therefore, CD4 + helper and CD8 + T-cells are more susceptible to glucose availability compared with Tregs. Moreover, highly glycolytic tumor cells promote an acidic TME by secreting lactate, a byproduct of glycolysis.

Lactic acid inhibits the survival and effector function of T-cells [33]. Lactic metabolites also inhibit CD4 + and CD8 + T-cell mobility [34]. Similarly, knockdown of the lactate-producing enzyme lactate dehydrogenase A promoted CD8 + T-cell infiltration in the TME while decreasing the number of Tregs in mice tumors. In addition, neutralizing the TME increased CD8 + T-cell effector functions and infiltration and reinforced the response to immunotherapy in mouse tumor models [35–37]. Therefore, increased tumor glycolysis can modulate the immune profile of the TME by restricting the availability of glucose to immune cells and secreting immunosuppressive lactic acid. Consistently, this study confirmed the inverse correlation between HK2 tumor expression and the ratio of CD8 + T-cells to Tregs in human lung cancer and colon cancer tissues.

The evaluation of Treg infiltration as a prognostic marker has shown conflicting results, which might be due to higher Treg infiltration in an inflamed TME rich in CD8 + T-cells and other immune cells [29]. In this study, increased Treg infiltration was associated with a favorable prognosis, which is a paradoxical finding given their immunosuppressive (i.e., pro-tumoral) effects (Supplementary Figure S3). This might be because Treg infiltration was positively correlated with CD8 + T-cell infiltration. Therefore, some researchers have evaluated the prognostic significance of Treg infiltration compared with CD8 + T-cell infiltration (e.g., the ratio of Tregs to CD8 + T-cells or the ratio of CD8 + T-cells to Tregs), rather than Treg infiltration alone [38–40].

In this study, a high number of CD8 + TILs or a high ratio of CD8 + T-cells to Tregs was associated with a favorable prognosis. In contrast, a low ratio of CD8 + T-cells to Tregs was consistently associated with a poor prognosis in patients with early- and advanced-stage lung SqCC. Although the statistical significance was marginal, a high CD8 + T-cell to Treg ratio was associated with better prognosis in lung cancer patients treated with immunotherapy. These findings reflect the biological roles of CD8 + T-cells and Tregs in anti-tumor immunity and emphasize the value of ratio of CD8 + T-cell to Treg ratio as a prognostic biomarker as well as a potential predictive biomarker. However, the implications of the CD8 + T-cell to Treg ratio as a predictive biomarker for immunotherapy need further study.

Tumor cells rely on increased metabolism, especially glycolysis for their survival, growth, and proliferation. This metabolic reprogramming also increases the aggressiveness of the tumor indirectly by modulating the TME into a pro-tumorigenic milieu. Therefore, attempts have been made to target tumor metabolism to enhance treatment responses and patient outcomes [41, 42]. HK2 is an interesting target for modulating metabolism. Several HK2 inhibitors have been evaluated in vitro and in mouse models, but no successful agents that clinically suppress tumor HK2 have been discovered due to their low specificity to tumor cells [43]. Even though there are more hurdles to overcome for developing clinically applicable HK2 inhibitors, there could be promising combination agents to enhance immunotherapy or chemotherapy responses by releasing immune cells from metabolic competition and directly decreasing tumor cell survival.

In summary, this study demonstrated that tumor HK2 expression/glycolysis can influence the immune profile of the TME, and an increase in tumor HK2 expression/glycolysis might be linked to a decreased CD8 + T-cell to Treg ratio, implying a poor prognosis. Although we did not investigate a detailed causal relationship between glycolysis and the TME, the above findings suggest that tumor metabolism may influence the tumor immune microenvironment into becoming pro- or anti-tumorigenic.

## Conclusion

An increase in tumor HK2 expression/glycolysis may contribute to shaping the immunosuppressive/pro-tumorigenic tumor immune microenvironment by modulating/decreasing the ratio of tumor-infiltrating CD8 + T-cells to regulatory T-cells in human cancers. Thus, tumor HK2 expression/glycolysis should be further investigated as potential biomarkers or therapeutic targets in the era of cancer immunotherapy.

## Supplementary Information

**Additional file 1:** **Supplementary Data S1.** Clinicopathological features and immunoprofiles of patients with lung adenocarcinoma subjected to flow cytometry. **Supplementary Data S2.** Clinicopathological features and immune profiles of patients with lung adenocarcinoma subjected to immunohistochemistry. **Supplementary Data S3.** Clinicopathological features and immune profiles of patients with lung squamous cell carcinoma subjected to immunohistochemistry. **Supplementary Data S4.** Clinicopathological features and immune profiles of patients with colon adenocarcinoma subjected to immunohistochemistry. **Supplementary Data S5.** Clinicopathological features and immune profiles of non-small cell lung cancer patients with PD-1/PD-L1 blockade subjected to immunohistochemistry.

**Additional file 2:** **Supplementary Table S1.** Clinicopathological features of patients with lung adenocarcinoma subjected to flow cytometry for comprehensive immunoprofiling. **Supplementary Table S2.** Clinicopathological features of patients with lung adenocarcinoma subjected to immunohistochemistry. **Supplementary Table S3.** Clinicopathological features of patients with lung squamous cell carcinoma subjected to immunohistochemistry. **Supplementary Table S4. **Clinicopathological features of patients with colon adenocarcinoma subjected to immunohistochemistry. **Supplementary Table S5.** Clinicopathological features of non-small cell lung cancer patients with PD-1/PD-L1 blockade subjected to immunohistochemistry. **Supplementary Table S6.** HK2 expression according to clinicopathologic parameters in lung adenocarcinoma patients. **Supplementary Table S7.** HK2 expression according to clinicopathologic parameters in lung squamous cell carcinoma patients. **Supplementary Table S8.** HK2 expression according to clinicopathologic parameters in colorectal cancer patients.

**Additional file 3:** **Supplementary Figure S1.** Gating strategies for lymphoid and myeloid cells in lung adenocarcinoma. **Supplementary Figure S2.** Correlation between CD8+ T-cell infiltration and Treg infiltration in human cancer tissues. **Supplementary Figure S3.** Survival analysis of  patients with lung SqCC according to the number of Tregs.

**Additional file 4.** Supplementary Methods.

## Data Availability

The datasets analyzed during the current study are available in Additional file 1.

## Abbreviations

ADC: Adenocarcinoma
AJCC: American Joint Committee on Cancer
CR: Complete response
CRC: Colorectal carcinoma
FAO: Fatty acid oxidation
HK2: Hexokinase-2
HNPCC: Hereditary nonpolyposis colorectal cancer
MD: Moderately differentiated
MDSC: Myeloid-derived suppressor cell
MSI-L or H: Microsatellite instability-low or high
MSS: Microsatellite stable
NA: Not assessable
OS: Overall survival
OXPHOS: Oxidative phosphorylation
PD: Poorly differentiated or Progressive disease
PFS: Progression free survival
PR: Partial response
PY: Pack-year
SD: Stable disease
SqCC: Squamous cell carcinoma
TAM: Tumor-associated macrophage
TIL: Tumor-infiltrating lymphocyte
TME: Tumor microenvironment
Treg: Regulatory T-cell
WD: Well differentiated
