# Supplementary material for: High tumor hexokinase-2 expression promotes a pro-tumorigenic immune microenvironment by modulating CD8+/regulatory T-cell infiltration
Source: BMC Cancer. 2022 Nov 1;22:1120. doi: 10.1186/s12885-022-10239-6 (PMC9628070; doi:10.1186/s12885-022-10239-6)
Supplement: Supplementary file 2 — Additional file 2: Supplementary Table S1. Clinicopathological features of patients with lung adenocarcinoma subjected to flow cytometry for comprehensive immunoprofiling. Supplementary Table S2. Clinicopathological features of patients with lung adenocarcinoma subjected to immunohistochemistry. Supplementary Table S3. Clinicopathological features of patients with lung squamous cell carcinoma subjected to immunohistochemistry. Supplementary Table S4. Clinicopathological features of patients with colon adenocarcinoma subjected to immunohistochemistry. Supplementary Table S5. Clinicopathological features of non-small cell lung cancer patients with PD-1/PD-L1 blockade subjected to immunohistochemistry. Supplementary Table S6. HK2 expression according to clinicopathologic parameters in lung adenocarcinoma patients. Supplementary Table S7. HK2 expression according to clinicopathologic parameters in lung squamous cell carcinoma patients. Supplementary Table S8. HK2 expression according to clinicopathologic parameters in colorectal cancer patients. [file 12885_2022_10239_MOESM2_ESM.docx]

**Supplementary Table S1. Clinicopathological features of patients with lung adenocarcinoma subjected to flow cytometry for comprehensive immunoprofiling**

| Clinicopathological parameter | N (Total = 41) (%) |
| --- | --- |
| Median age at diagnosis (range in years) | 67 (48−88) |
| Sex |  |
| Male | 21 (51.2) |
| Female | 20 (48.8) |
| Smoking |  |
| No | 25 (61.0) |
| Yes | 16 (39.0) |
| T stage |  |
| T1 | 9 (21.9) |
| T2 | 29 (70.8) |
| T3 | 2 (4.9) |
| N stage |  |
| N0 | 31 (75.6) |
| N1−2 | 10 (24.4) |
| *EGFR* mutation* |  |
| Wild-type | 18 (47.4) |
| Mutated | 20 (52.6) |
| *ALK* translocation* |  |
| Not translocated | 38 (100) |
| Translocated | 0 |

*Some cases have missing values.

Abbreviations: ALK, anaplastic lymphoma kinase; EGFR, epidermal growth factor receptor

**Supplementary Table S2. Clinicopathological features of patients with lung adenocarcinoma subjected to immunohistochemistry**

| Clinicopathological parameter | N (Total = 375) (%) |
| --- | --- |
| Median age at diagnosis (range in years) | 64 (27−86) |
| Sex |  |
| Female | 189 (50.4) |
| Male | 186 (49.6) |
| Smoking |  |
| No | 234 (62.4) |
| Yes | 141 (37.6) |
| T stage* |  |
| T1 | 184 (49.1) |
| T2 | 161 (42.9) |
| T3 | 22 (5.9) |
| T4 | 4 (1.3) |
| N stage* |  |
| N0 | 303 (81.5) |
| N1 | 25 (6.7) |
| N2 | 42 (11.3) |
| N3 | 2 (0.5) |
| M stage* |  |
| M0 | 370 (98.7) |
| M1 | 5 (1.3) |
| AJCC 7^th^ stage* |  |
| I | 277 (74.1) |
| II | 43 (11.5) |
| III | 49 (13.1) |
| IV | 5 (1.3) |
| *EGFR** |  |
| Wild-type | 137 (38.4) |
| E19del | 119 (33.3) |
| L858R | 78 (21.8) |
| Other | 23 (6.4) |
| *ALK** |  |
| Wild | 351 (98.3) |
| Translocation | 6 (1.6) |
| *KRAS** |  |
| Wild | 331 (93.2) |
| Mutant | 24 (6.8) |

*Some cases have missing values.

Abbreviations: ALK, anaplastic lymphoma kinase; EGFR, epidermal growth factor receptor; KRAS, Kirsten rat sarcoma virus

**Supplementary Table S3. Clinicopathological features of patients with lung squamous cell carcinoma subjected to immunohistochemistry**

| Clinicopathological parameter | N (Total = 118) (%) |
| --- | --- |
| Median age at diagnosis (range in years) | 67 (43−87) |
| Sex |  |
| Female | 7 (5.9) |
| Male | 111 (94.1) |
| Smoking |  |
| No | 10 (8.5) |
| Yes | 108 (90.5) |
| T stage |  |
| T1 | 20 (17.0) |
| T2 | 58 (57.6) |
| T3 | 23 (19.5) |
| T4 | 7 (5.9) |
| N stage* |  |
| N0 | 64 (55.2) |
| N1 | 32 (27.6) |
| N2 | 20 (17.2) |
| AJCC 7^th^ stage* |  |
| I | 39 (33.6) |
| II | 46 (39.7) |
| III | 31 (26.7) |

*Some cases have missing values.

**Supplementary Table S4. Clinicopathological features of patients with colon adenocarcinoma subjected to immunohistochemistry**

| Clinicopathological parameter | N (Total = 338) (%) |
| --- | --- |
| Median age at diagnosis (range in years) | 22−85 (median, 60) |
| Sex |  |
| Female | 132 (39.1) |
| Male | 206 (60.9) |
| Diagnosis |  |
| Conventional CRC | 334 (98.8) |
| HNPCC | 4 (1.2) |
| MSI* |  |
| MSS | 294 (91.6) |
| MSI-L | 1 (0.3) |
| MSI-H | 26 (8.1) |
| Differentiation* |  |
| WD | 47 (13.9) |
| MD | 271 (80.4) |
| PD | 19 (5.6) |
| T stage |  |
| T1 | 27 (8.0) |
| T2 | 42 (12.4) |
| T3 | 254 (75.1) |
| T4 | 15 (4.4) |
| N stage |  |
| N0 | 204 (60.4) |
| N1 | 95 (28.1) |
| N2 | 39 (11.5) |
| M stage |  |
| M0 | 297 (87.9) |
| M1 | 41 (12.1) |
| AJCC 8th |  |
| I | 60 (17.8) |
| II | 138 (40.8) |
| III | 99 (29.3) |
| IV | 41 (12.1) |
| LVI |  |
| Not identified | 246 (72.8) |
| Present | 92 (17.2) |
| Neural invasion |  |
| Not identified | 257 (76.0) |
| Present | 81 (24) |

Abbreviations: CRC, colorectal cancer; HNPCC, hereditary nonpolyposis colorectal cancer; MSI, microsatellite instability; WD, well differentiated; MD, moderately differentiated; PD, poorly differentiated; LVI, lymphovascular invasion

**Supplementary Table S5. Clinicopathological features of non-small cell lung cancer patients with PD-1/PD-L1 blockade subjected to immunohistochemistry**

| Clinicopathological parameter | N (Total = 78) (%) |
| --- | --- |
| Median age at diagnosis (range in years) | 63 (33−88) |
| Sex |  |
| Male | 62 (79.5) |
| Female | 16 (20.5) |
| Smoking |  |
| No | 25 (32.1) |
| Yes | 53 (67.9) |
| Histology |  |
| Adenocarcinoma | 44 (56.4) |
| Squamous cell carcinoma | 23 (29.5) |
| Others | 11 (14.1) |
| Treatment modalities |  |
| Nivolumab | 33 (42.3) |
| Pembrolizumab | 24 (30.8) |
| Atezolizumab | 19 (24.4) |
| Durvalumab | 2 (2.6) |
| Best response to ITx* |  |
| CR/PR | 23 (32.4) |
| SD | 24 (33.8) |
| PD | 24 (33.8) |

*Some cases have missing values.

Abbreviations: CR, complete response; PR, partial response; SD, stable disease; PD, progressive disease

**Supplementary Table S6. HK2 expression according to clinicopathologic parameters in lung adenocarcinoma patients**

| Clinicopathological parameter | HK2 (H-score, mean) | *P* |
| --- | --- | --- |
| Age at diagnosis | Spearman rho, 0.015 | 0.768 |
| Sex |  | 0.964 |
| Female | 148.36 |  |
| Male | 148.39 |  |
| Smoking |  | 0.686 |
| No | 149.7 |  |
| Yes | 146.17 |  |
| T stage* |  | 0.306 |
| T1 | 154.13 |  |
| T2 | 141.06 |  |
| T3 | 156.82 |  |
| T4 | 124 |  |
| N stage* |  | 0.105 |
| N0 | 151.91 |  |
| N1 | 123.60 |  |
| N2 | 136.19 |  |
| N3 | 150 |  |
| M stage* |  | 0.924 |
| 0 | 148.35 |  |
| 1 | 150 |  |
| AJCC 7^th^ stage* |  | 0.606 |
| I | 151.19 |  |
| II | 145.12 |  |
| III | 136.12 |  |
| IV | 150 |  |
| *EGFR** |  | 0.079 |
| Wil-type | 153.65 |  |
| Mutant | 142.36 |  |
| *ALK** |  | 0.948 |
| Wild-type | 146.58 |  |
| Translocation | 153.33 |  |
| *KRAS** |  | 0.253 |
| Wil-type | 145.50 |  |
| Mutant | 162.50 |  |

*Some cases have missing values.

Abbreviations: ALK, anaplastic lymphoma kinase; EGFR, epidermal growth factor receptor; KRAS, Kirsten rat sarcoma virus

**Supplementary Table S7. HK2 expression according to clinicopathologic parameters in lung squamous cell carcinoma patients**

| Clinicopathological parameter | | HK2 (H-score, mean) | | *P* |
| --- | --- | --- | --- | --- |
| Age at diagnosis | Spearman rho, −0.057 | | 0.539 | |
| Sex |  | | 0.165 | |
| Female | 150 | |  | |
| Male | 195.41 | |  | |
| Smoking |  | | 0.235 | |
| No | 167 | |  | |
| Yes | 195.09 | |  | |
| T stage |  | | 0.427 | |
| T1 | 217 | |  | |
| T2 | 184.71 | |  | |
| T3 | 193.48 | |  | |
| T4 | 198.57 | |  | |
| N stage* |  | | 0.556 | |
| N0 | 188.13 | |  | |
| N1 | 192.19 | |  | |
| N2 | 206 | |  | |
| AJCC 7^th^ stage* |  | | 0.983 | |
| I | 191.28 | |  | |
| II | 190 | |  | |
| III | 197.10 | |  | |

*Some cases have missing values.

**Supplementary Table S8. HK2 expression according to clinicopathologic parameters in colorectal cancer patients**

| Clinicopathological parameter | HK2 (H-score, mean) | *P* |
| --- | --- | --- |
| Age | Spearman rho, −0.039 | 0.474 |
| Sex |  | 0.215 |
| Male | 173.71 |  |
| Female | 165.93 |  |
| Diagnosis |  | 0.374 |
| Conventional CRC | 168.75 |  |
| HNPCC | 187.50 |  |
| MSI |  | 0.447 |
| MSS | 169.57 |  |
| MSI-L | 250 |  |
| MSI-H | 168.65 |  |
| Differentiation |  | 0.974 |
| WD | 165.75 |  |
| MD | 169.35 |  |
| PD | 172.63 |  |
| pT |  | 0.070 |
| 1 | 156.48 |  |
| 2 | 172.26 |  |
| 3 | 168.08 |  |
| 4 | 197.33 |  |
| pN |  | 0.514 |
| 0 | 171.32 |  |
| 1 | 164.63 |  |
| 2 | 167.24 |  |
| pM |  | 0.275 |
| 0 | 169.68 |  |
| 1 | 163.84 |  |
| AJCC 8th |  | 0.176 |
| I | 166.25 |  |
| II | 176.77 |  |
| III | 161.87 |  |
| IV | 163.84 |  |
| LVI |  | 0.649 |
| Not identified | 170.57 |  |
| Present | 164.70 |  |
| Neural invasion |  | 0.506 |
| Not identified | 168.46 |  |
| Present | 170.59 |  |

Abbreviations: CRC, colorectal cancer; HNPCC, hereditary nonpolyposis colorectal cancer; MSI, microsatellite instability; WD, well differentiated; MD, moderately differentiated; PD, poorly differentiated; LVI, lymphovascular invasion
