# Supplementary material for: High tumor hexokinase-2 expression promotes a pro-tumorigenic immune microenvironment by modulating CD8+/regulatory T-cell infiltration
Source: BMC Cancer. 2022 Nov 1;22:1120. doi: 10.1186/s12885-022-10239-6 (PMC9628070; doi:10.1186/s12885-022-10239-6)
Supplement: Supplementary file 3 — Additional file 3: Supplementary Figure S1. Gating strategies for lymphoid and myeloid cells in lung adenocarcinoma. Supplementary Figure S2. Correlation between CD8+ T-cell infiltration and Treg infiltration in human cancer tissues. Supplementary Figure S3. Survival analysis of patients with lung SqCC according to the number of Tregs. [file 12885_2022_10239_MOESM3_ESM.pdf]

Supplementary Figure S1.

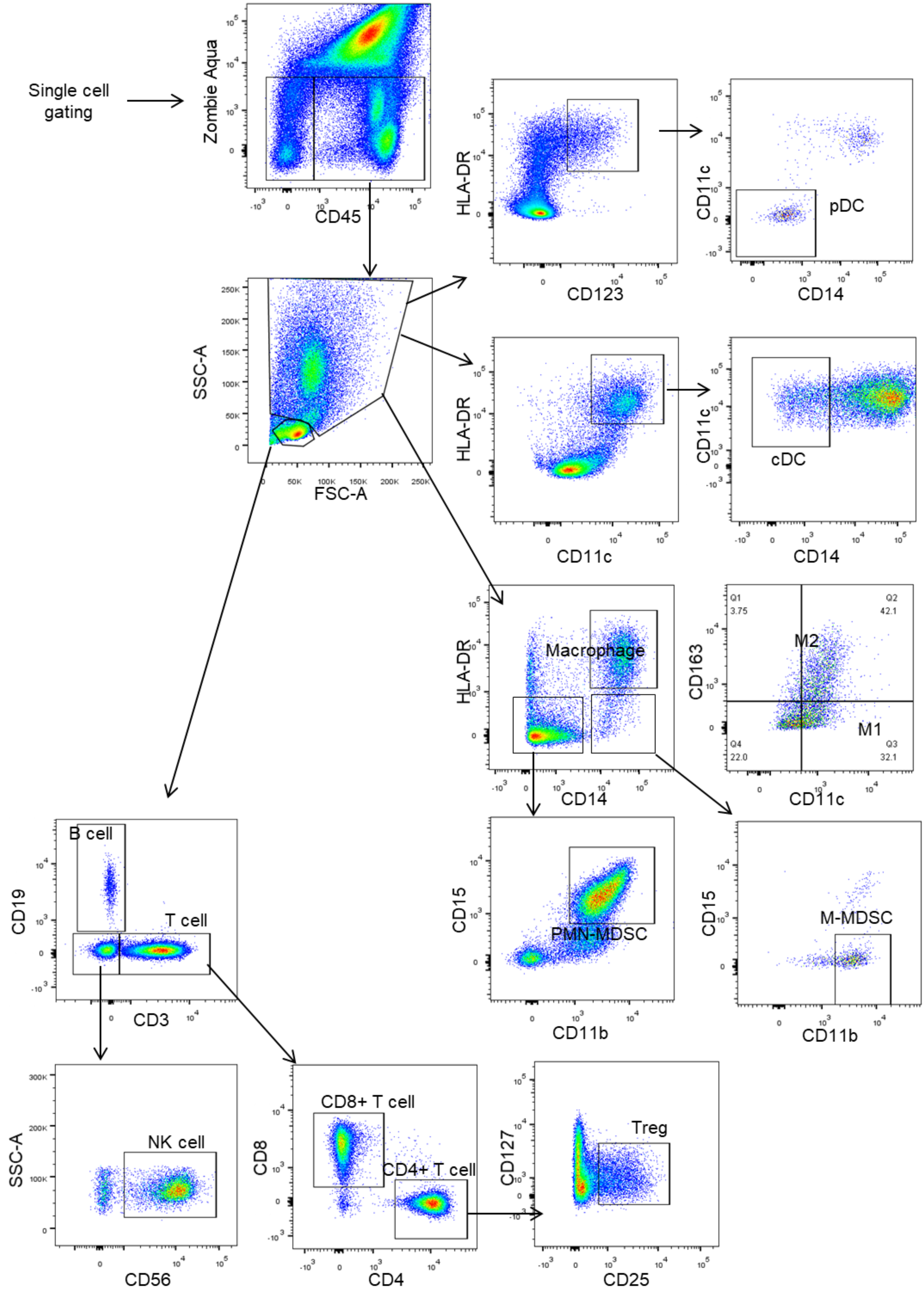

Supplementary Figure S1. Gating strategies for lymphoid and myeloid cells in lung adenocarcinoma

Supplementary Figure S2.

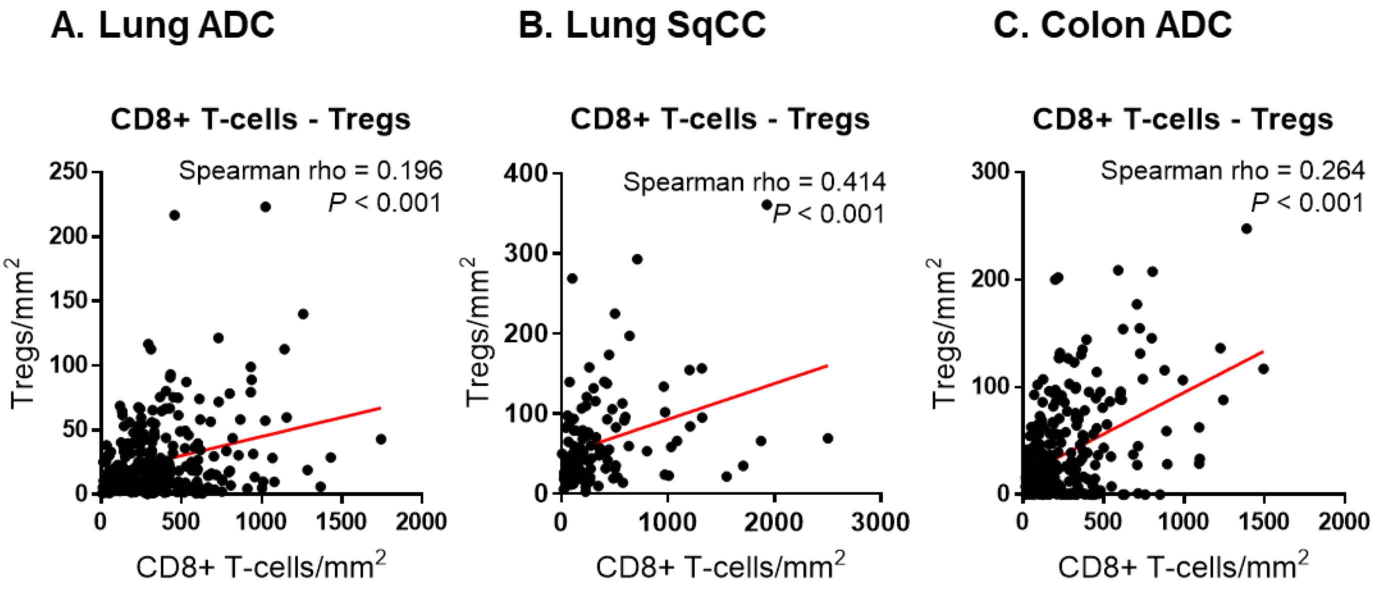

Supplementary Figure S2. Correlation between CD8+ T-cell infiltraion and Treg infiltration in human cancer tissues. The positive correlation between CD8+ T-cell and Treg infiltration is observed in lung ADC **(A)**, lung SqCC **(B)** and colon ADC **(C)**. All *P* values were calculated using Spearman correlation analysis.

Supplementary Figure S3.

Tregs

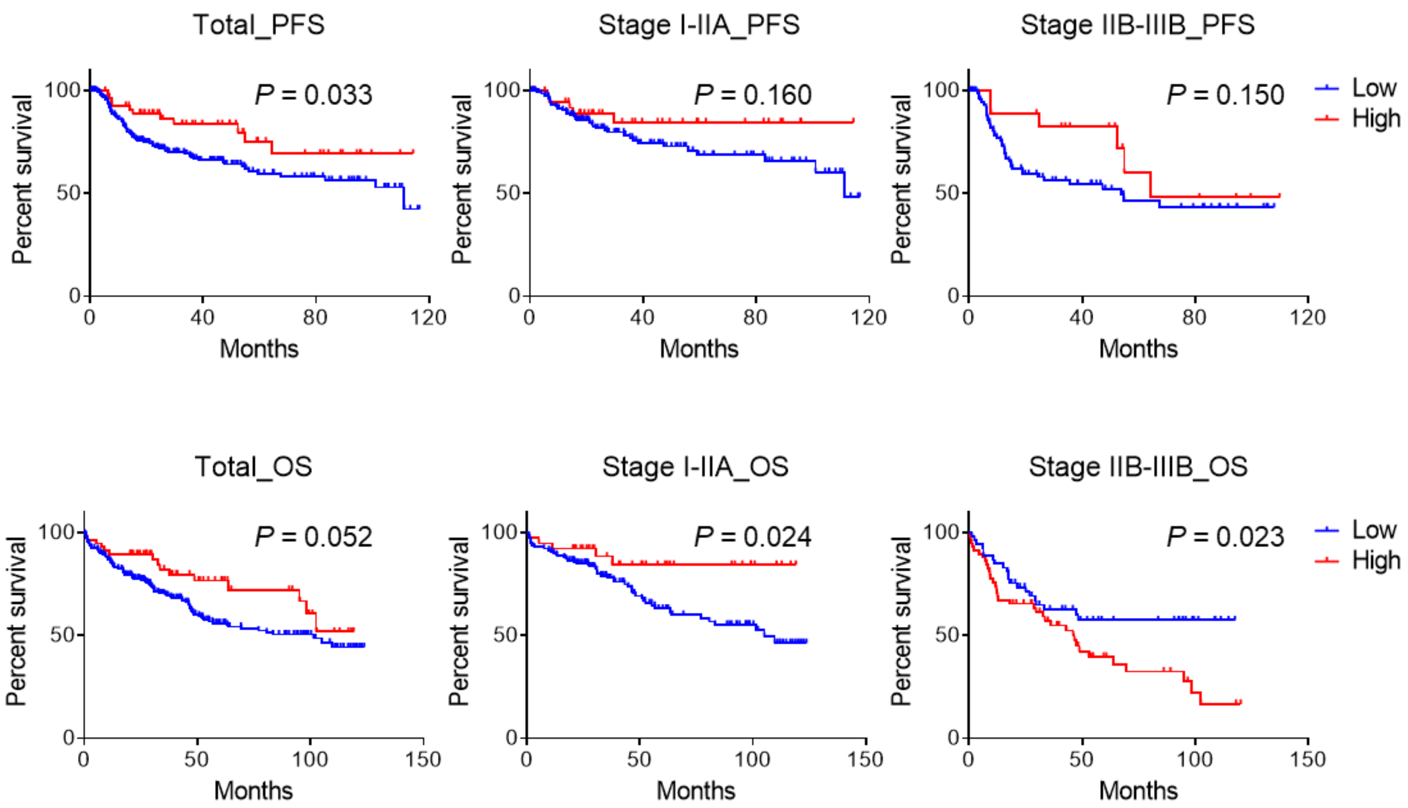

Supplementary Figure S3. Survival analysis of patients with lung SqCC according to the number of Tregs. The progression-free survival **(A)** and overall survival **(B)** according to the number of Tregs are displayed in total, early-stage (stage I–IIA), and advanced-stage (stage IIB–IIIB) lung SqCC patients. The survival difference was plotted and analyzed using the Kaplan-Meier and log-rank test.
