# Supplementary material for: High tumor hexokinase-2 expression promotes a pro-tumorigenic immune microenvironment by modulating CD8+/regulatory T-cell infiltration
Source: BMC Cancer. 2022 Nov 1;22:1120. doi: 10.1186/s12885-022-10239-6 (PMC9628070; doi:10.1186/s12885-022-10239-6)
Supplement: Supplementary file 4 — Additional file 4. Supplementary Methods. [file 12885_2022_10239_MOESM4_ESM.docx]

**Supplementary Methods**

To analyzing tumor-infiltrating immune cell subsets, about 1 gram of fresh lung ADC tissues were minced using sterile blades, digested in RPMI-1640 supplemented with 80 U/ml DNase I, 300 U/ml collagenase I and 60 U/ml hyaluronidase at 37 °C for 30 min, and then filtered using a 70 μm cell strainer. After red blood cell lysis, cells were then stained for dead cell exclusion using an amine-reactive dye (Aqua LIVE/DEAD Stain Kit; Life Technologies, Thermo Fisher Scientific, Carlsbad, CA, USA) and then, pre-incubated with Fc receptor blocking solution (BioLegend, San Diego, CA, USA) to reduce non-specific binding. Cells were stained using the appropriate antibodies in FACS buffer for at least 30 min at 4℃ in dark. The panels for lymphoid cell and myeloid cell analysis are listed below.

| **Target molecules** | **Fluorochrome** | **Clone, company** |
| --- | --- | --- |
| **Lymphoid cells** | | |
| CD45 | Alexa 700 | Hi30; BioLegend |
| CD3 | Percp-Cy5.5 | UCHT1; BD biosciences |
| CD4 | Alexa 488 | RPA-T4; BioLegend |
| CD8 | APC-cy7 | SK1; BioLegend |
| CD19 | BV421 | HIB19; BioLegend |
| CD56 | BV605 | NCAM16.2; BD biosciences |
| CD25 | BV711 | 2A3; BD biosciences |
| CD127 | PE-Cy7 | A019D5; BioLegend |
| **Myeloid cells** | | |
| CD45 | Alexa 700 | Hi30; BioLegend |
| CD14 | Alexa 488 | MQP9; BD biosciences |
| HLA-DR | BV786 | G46-6; BD biosciences |
| CD11c | PE-cy7 | B-ly6; BD biosciences |
| CD123 | BV650 | 7G3; BD biosciences |
| CD163 | BV421 | GHI/61; Biolegend |
| CD15 | PE | SSEA-1; Biolegend |
| CD11b | APC-cy7 | M1/70; Biolegend |
